# Oral health status and its associated factors among post-stroke inpatients: a cross-sectional study in Hungary

**DOI:** 10.1186/s12903-022-02259-2

**Published:** 2022-06-14

**Authors:** Júlia Moldvai, Mercédesz Orsós, Eszter Herczeg, Eszter Uhrin, Márton Kivovics, Orsolya Németh

**Affiliations:** 1 Department of Community Dentistry, Faculty of Dentistry, Semmelweis University, Szentkirályi str. 40, Budapest, 1088 Hungary; 2 Department of Rehabilitation Post-Stroke, National Institute for Medical Rehabilitation, Budapest, Hungary

**Keywords:** Stroke, Oral health status, Dental caries, Stroke rehabilitation, Functional assessment

## Abstract

**Background:**

Post-stroke inpatients are at risk of poor oral health for a number of reasons. The aim of this study was to assess the oral health status of post-stroke patients and also to explore the factors that may influence it.

**Methods:**

This cross-sectional study was organised at National Institute for Medical Rehabilitation in Hungary. Altogether 410 post-stroke patients were enrolled in the survey. Personal medical history and functional assessment was obtained from the final medical reports of the patients. The clinical examination and data collection were conducted according to the World Health Organization (2013) criteria. Socio-demographic background and behaviours related to oral health were assessed using a questionnaire. The dental status was explained by the number of Decayed, Missing and Filled Teeth (DMFT). The association of socio-demographic factors, stroke and functional assessment with oral health status and behaviour was evaluated. Chi-square test, Fisher’s exact test, Welch test, Mann–Whitney U test, Kruskal–Wallis test, ANOVA model and correlation analysis were used to analyse our data. The level of significance was set at *p* < 0.05.

**Results:**

Mean age of stroke patients was 59.21(Standard Deviation [SD] 14.74) years. Mean DMFT score was 20.13 (8.08), including 3.28 (4.24) decayed teeth, 15.02 (10.29) missing teeth and 1.83 (2.94) filled teeth score. Factors that influenced the oral health status were gender, age, occupational status, level of education, type and risk factors for stroke. Significant correlation was found between the Functional Independence Measure and oral health-related behaviours with patients brushing their teeth once a month showing the lowest value.

**Conclusion:**

According to the results, low socio-demographic and economic status, low level of education and the Functional Independence Measure score, unemployment, the combination of risk factors for stroke and residual dysfunctions are associated with poor oral health status. The data indicate that a series of changes are needed, for special attention and care in oral health for patients who have had a stroke. Based on the findings of this research, a new model of prevention and care can be developed, with an interdisciplinary collaboration, to promote the quality of life of these individuals.

**Supplementary Information:**

The online version contains supplementary material available at 10.1186/s12903-022-02259-2.

## Background

Stroke is a serious cerebrovascular disease and the third most common cause of death in the world after coronary heart diseases and cancer [1]. Nearly 15 million people suffer a stroke worldwide each year, of those 5 million die and 5 million continue to live with a disability [1]. In Hungary, the data from the Hungarian Central Statistical Office report an increasing number of cases from year to year, e.g. in 2019 more than 760 cases were reported per 10,000 people [2].

The major modifiable risk factors for stroke are hypertension, diabetes mellitus, hyperlipidemia, tobacco smoking and alcohol consumption [3]. Higher age, male gender and low educational level increase the risk of stroke as from predisposing comorbidities [4]. In addition, age, gender, education level, occupational status, diabetes, smoking, alcohol consumption are some of the common risk factors with poor oral health status.

In many countries around the world, several studies have examined the cause and effect relationship between oral health and stroke [5–7]. Systemic diseases may have oral manifestations and on the other hand poor oral hygiene may affect the general condition and quality of life. Previous studies have reported that post-stroke patients had worse oral health status across a range of parameters (tooth loss, dental caries, periodontal status, prosthetic index) compared with the healthy group [8–10]. However, Hungarian research reports about the oral health status of post-stroke patients are limited. There is just one Hungarian study in the literature reporting on the oral health status of post-stroke patients [8].

Post-stroke patients undergoing rehabilitation are at risk of poor oral health for a number of reasons. The long-term effects of cerebral damage such as restricted physical abilities, cognitive deficits, lack of coordination, neuropsychological sequels, plus mental health problems make it difficult to maintain good oral health. Post-stroke symptoms like hemiparesis (one-sided weakness), hemiplegia (one-sided paralysis), lack of sensation, apraxia (inability to perform a skilled motor activity), dysarthria (motor speech disorder), swallowing dysfunction, and ataxia (lack of muscle control or coordination of voluntary movements) constitute a substantial challenge in the daily oral care routine and dental visits [11]. Aphasia and agnosia can cause considerable problems in communication between patient and dentist [8]. Stroke can cause impairment of the masticatory muscles, lips, tongue, soft palate and pharynx, which may impact speaking, food intake and oral clearance. The trapped food debris will result in tooth decay, halitosis and an increased risk of other microbial infections [12]. Moreover, poor oral health contributes to an increased number of bacteria in the saliva, which when aspirated can lead to pneumonia [13]. These complications interfere with the progress of the rehabilitation treatment [14].

Oral hygiene is often neglected due to a patients’ physical or cognitive difficulties. In this way, patients are reliant on nursing staff and caregivers to maintain their good oral hygiene [15]. Oral care could be improved with more training opportunities for nursing staff and caregivers, assessing patients’ remaining abilities on admission, and using protocols to guide proper oral care [16].

Post-stroke patients require special oral care. Oral health care and dental rehabilitation have to be a part of the general rehabilitation for post-stroke inpatients [17].

In 2015, the Department of Community Dentistry of Semmelweis University and The National Institute for Medical Rehabilitation (NIMR) began to collaborate and opened a dental practice to gain comprehensive information on patients’ oral health and oral hygiene practices undergoing rehabilitation [18, 19].

The aim of this study was to assess the oral health status and behaviours of patients who are undergoing rehabilitive treatment after stroke and to assess the association with socio-demographic factors, stroke-related factors, functional status and oral health-related behaviours.

## Methods

### Study design

This cross-sectional study was organised at NIMR in Hungary. Examination and data collection were performed from August 2016 to September 2020. This research has been approved by the Medical Research Council, Scientific and Research Committee of Hungary (No: ETT-TUKEB IV/1433–1/2020/EKU), and was performed according to the Declaration of Helsinki [20]. All patients provided written consent for participation. The study followed the recommendations of STROBE (Strengthening the Reporting of Observational Studies in Epidemiology).

### Study size

The sample size was determined based on the results of previous cross-sectional study in this subject area using the G*Power 3.1 software (v.3.1.9.3, 2017, Institut für Experimentelle Psychologie, Heinrich-Heine-Universität, Düsseldorf, Germany). Our calculation was based on the results of Orsós et al. [21] according to which if *α* (false positive rate) was set at 0.05, to reach a power of 95% with a 1:1 distribution ratio between study groups the minimal sample size should be at least 97 (association between smoking and dental status) and 132 (association between alcohol consumption and dental status) per study group.

### Patients

410 post-stroke patients (174 female, 236 male), whose primary diagnosis was subarachnoid hemorrhage, intracerebral hemorrhage or cerebral infarction (International Classification of Diseases, 10 th revision (ICD-10): I60x, I61x, I63x, I67x) were included in this study. In processing the data, the two main types of hemorrhagic stroke (intracerebral and subarachnoid) were examined separately according to the International Classification of Diseases recommended by the World Health Organization (WHO) [22]. All of the stroke patients were recruited while receiving rehabilitative care at NIMR. Inclusion criteria were medical stability and confirmed stroke diagnosis. Inpatients were excluded if they had had nasogastric tube placement or if they indicated communication difficulties (unable to follow a 1-step command) or if they had recurrent stroke.

### Personal medical history and functional assessment

On admission, the medical reports of the patients were collected, the history, the initial diagnosis, the type of stroke (subarachnoid or intracerebral and cerebral infarction), time since diagnosis, presence of risk factors for stroke (diabetes, hypertension, hyperlipidemia, atrial fibrillation, ischemic heart disease, heart failure), post-stroke sequelae [hemiplegia/hemiparesis, tetraplegia (paralysis in the upper and lower body), dysphagia (swallowing problems), dysarthria/anarthria (complete loss of speech motor ability), ataxia and depression], Functional Independence Measure (FIM) scores [23] and Barthel Index scores [24]. The FIM and the Barthel index are widely used outcome measures in neurological rehabilitation that assess the level of independence in activities of daily living [25]. The FIM is an 18-item tool that estimates function in 6 areas (self-care, including sphincter control, tranfers, mobility, communication, cognition) and measures functional disability of inpatients in terms of their need for assistance. Each item is scored from 1 to 7 and total FIM score range from 18 to 126 [26]. The Barthel index define the grade of the self-sufficiency inlcuding 10 personal activities. The total score for the Barthel index is a value between 0 and 100 [27].

### Socio-demographic factors

Socio-demographic background (age, gender, level of education, occupational status, location type) were obtained from a validated questionnaire and patient documentation. The patients’ age was classified as 17–24, 25–49, 50–64, 65–79, and 80–89 years. Gender was recorded as male or female. Level of education was categorised into seven groups: no formal schooling, less than primary school, primary school completed, secondary school completed, high school completed, college/university completed, postgraduate degree. Occupational status was recorded according to patients’ responses based on local opportunities, which were classified into three main groups according to the Hungarian Central Statistical Office [28], as active (employed, self-employed), inactive (student, housewife, stay at home parent, pensioner, altered work ability) and unemployed. Location type (permanent address) was categorised into three groups: capital, periurban area and rural.

### Behavioural factors

Dental health practices (last dental visit, tooth-brushing habits), frequency of smoking and alcohol consumption were assessed using a validated questionnaire that was based on the Oral health surveys: basic methods, 5th edition by WHO [29].

### Oral examination

The oral examination was carried out by two calibrated dentists using a plane mouth mirror, a dental probe and an artificial light. The examiners were trained and calibrated by an experienced dental epidemiologist. The calibrator examined 25 subjects who were also examined by two dentists. The diagnosis found by reference examiner was compared to those of the calibrated dentists. To assess the consistency of each individual examiner and the variations between examiners, each examiner first practised the examination on a group of 10 subjects. Every examiner then independently was examined the same group of 20 subjects and compared the findings with the other examiner. The kappa value of intra-examiner reproducibility was 0.85, while for inter-examiner reproducibility was 0.87. Diagnostical criteria and codes of dentition status were recorded according to the criteria of WHO [29]. Accordingly, a tooth was coded as sound if it shows no evidence of treated or untreated clinical caries. A tooth was diagnosed as decayed (code:1), if there was a lesion in a pit or fissure, or if on a smooth tooth surface had an unmistakable cavity, undermined enamel, or a detectably softened floor or wall. A tooth was recorded as filled with decay (code:2) when the crown had one or more restorations and one or more areas that were decayed. A crown was filled with no decay (code:3) when one or more restorations were present without caries. A missing tooth as a result of decay (code:4) or any other reasons (code:5) was used for teeth that have been extracted. The prevalence of caries was assessed based on Decayed, Missing and Filled Teeth Index (DMFT), which could be derived directly from the codes before. The D component (D-T) includes all teeth with codes 1 or 2. The M component (M-T) contains teeth coded 4 and 5. The F component (F-T) comprises teeth only with code 3. The wisdom teeth were included in the calculation, so the highest score was 32.

The type of prosthetic appliances (fixed prosthetic appliances, removable prosthetic appliances) and the number of prosthetically replaced teeth were also recorded. The number of prosthetically replaced teeth per person was evaluated using the Prosthetic Index, which was expressed in a percentage [30]. The level of dental care (restorative index) was expressed as [(F/D + F)*100] at tooth level, as in a previous study [18, 19]. The restorative index described the proportion of the decayed and filled teeth that had been treated restoratively.

### Data collection

All data was recorded on tablets based on an Android operating system. The data collection and analysis software were created in a collaboration with scientists from the Wigner Research Centre for Physics, located in Budapest. All the collected information was uploaded directly to the Hungarian Academy of Science’s cloud-based data storage. For statistical analysis, data could be retrieved from the cloud and converted to Microsoft Office Excel, version 2019.

### Statistical analysis

The analysis of data was performed using the statistical software R, version 4.0.2 (R Core Team, 2020, Vienna, Austria) using the coin (Hothorn, Hornik, van de Wiel and Zeileis, 2008), tidyverse (Wickham et al., 2019), knitr (Xie, 2021), RcmdrMisc (Fox, 2020), summarytools (Comtois, 2021), ggpubr (Kassambara, 2020) packages. For the descriptive analysis of categorical variables, case number and percentage were computed, while in the case of continuous variables, patient number, mean, Standard Deviation (SD), median, 25% and 75% quartiles (IQR) were calculated. The socio-demographic factors, stroke-related factors, functional assessment and oral health-related behaviour were examined for their association with the number of decayed, missing and filled teeth. The correlation between FIM and frequency of tooth cleaning was also investigated. To identify the association between dental status and various factors, we used the Chi-square test and Fisher’s exact test in case of categorical data, the Welch test for normally distributed variables, and the Mann–Whitney U test and Kruskal–Wallis test for non-normally distributed variables. Analysis of Variance technique by Blocks (ANOVA) was used to detect the statistical differences between three or more independent groups. Correlation analysis was used to measure linear relationships between FIM, Barthel index and D-T, M-T, F-T, DMFT score. The level of significance was set at *p* < 0.05. The total sample size for the study was 410, however, data was missing in 8 cases in FIM scores and 13 cases in Barthel Index scores. Patients with these missing data (8 patients) were excluded from the statistical calculations where association between functional status and dental status or frequency of tooth cleaning was calculated, however, they were included in all other calculations because patients weren’t treated as individual data but as groups.

## Results

### Study population

**Fig. 1:**
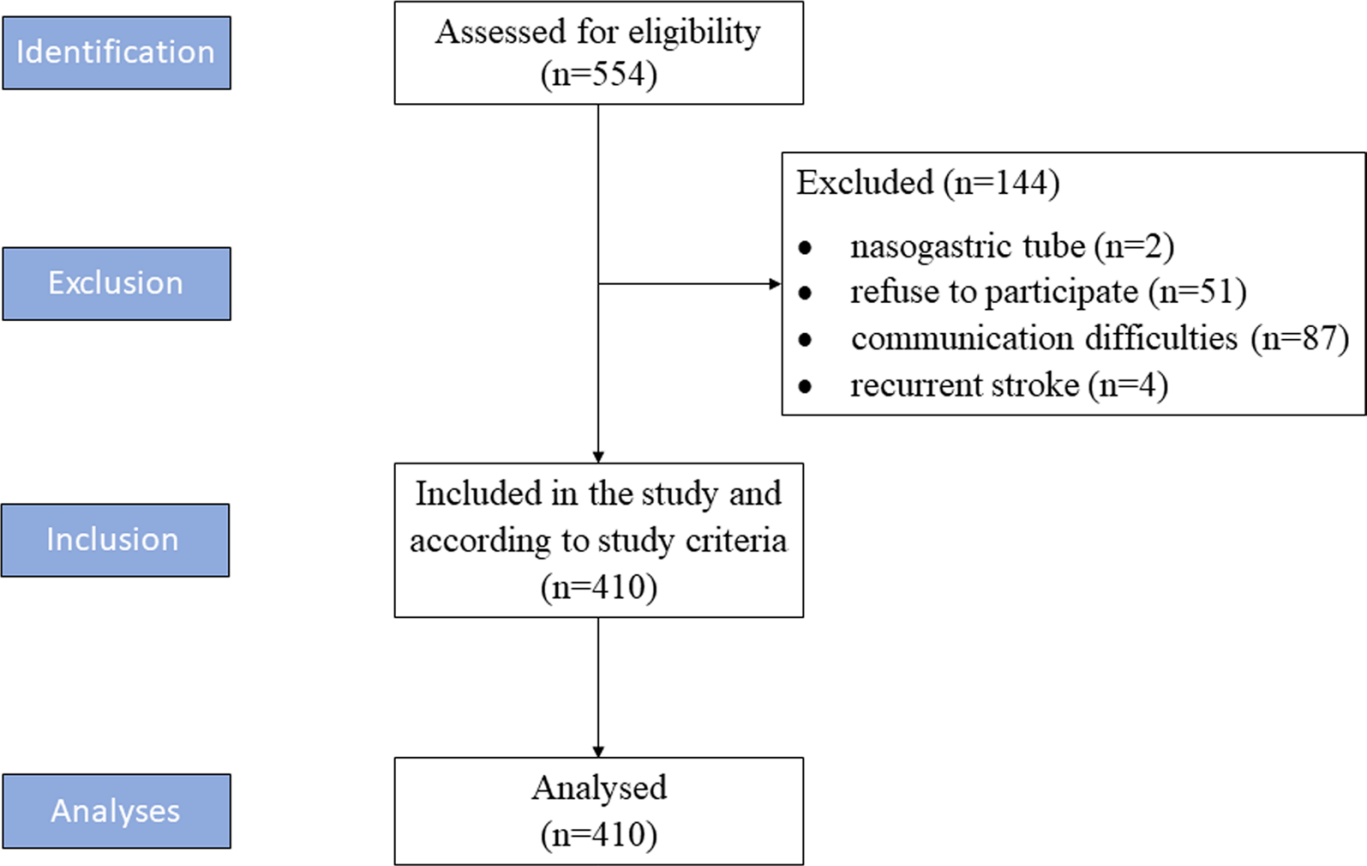
Flow diagram

During the study period, 410 (174 female, 236 male) out of 554 eligible patients were enrolled (Fig. 1). All of the post-stroke patients were Hungarian.

### Patient characteristics

Table 1 shows the patients’ socio-demographic and clinical characteristics at the time of admission.

**Table 1:** Patient characteristics

Characteristics	Overall *n* = 410
Gender, *n* (%)	
Male	236 (57.6)
Female	174 (42.4)
Age, years mean (SD)	58.72 (13.63)
Age, years, *n* (%)	
17–24 years	6 (1.5)
25–49 years	104 (25.4)
50–64 years	146 (35.6)
65–79 years	134 (32.7)
≥ 80 years	20 (4.9)
Location type, *n* (%)	
Capital	154 (37.6)
Periurban area	39 (9.5)
Rural	217 (52.9)
Occupational status, *n* (%)	
Active	217 (52.9)
Inactive	183 (44.6)
Unemployed	10 (2.4)
Level of education, *n* (%)	
No formal schooling	2 (0.5)
Less than primary school	5 (1.2)
Primary school completed	54 (13.2)
Secondary school completed	92 (22.4)
High school completed	126 (30.7)
College/university completed	130 (31.7)
Postgraduate degree	1 (0.2)
Stroke type, *n* (%)	
Hemorrhagic stroke	89 (21.7)
Cerebral infarction	321 (78.3)
Post-stroke sequels, *n* (%)	
Hemiplegia/hemiparesis	367 (89.5)
Tetraplegia/quadriplegia	11 (2.7)
Dysphagia	18 (4.4)
Dysphasia	61 (14.9)
Dysarthria/anarthria	33 (8.1)
Ataxia	39 (9.5)
Post-stroke depression	67 (16.3)
Risk factors for stroke, *n* (%)	
Diabetes	88 (21.5)
Hypertension	310 (75.6)
Hyperlipidemia	121 (29.5)
Atrial fibrillation	37 (9.0)
Ischemic heart disease	52 (12.7)
Heart failure	11 (2.7)
Alcohol consumption	272 (66.3)
Smoking	117 (28.5)
FIM at admission, mean (SD)	76.47 (29.5)
Barthel Index at admission, mean (SD)	49.55 (34.49)
Time since diagnosis of stroke (months), mean (SD)	10.07 (26.4)

*FIM* Functional Independence Measure, *SD* Standard Deviation

The mean age was 59.21 (Standard Deviation [SD] 14.74) years for female patients, and 58.36 (12.77) years for male patients. According to the distribution by residence, more than half of inpatients had a rural address (52.9%). More than half of the participants in the research were actively employed (52.9%), with most patients having graduated from college or university. In more than three-quarters of the cases, the stroke type was cerebral infarction (78.3%). The most common post-stroke symptom in patients that may have been infered with dental care was hemiplegia/hemiparesis. There were only 17 patients in whom none of the listed risk factors were found (4.1%).

### Oral health status

The mean DMFT score of the patients was 20.13 (Standard Deviation [SD] 8.08), including 3.28 (4.24) D-T, 15.02 (10.29) M-T and 1.83 (2.94) F-T score. Of the stroke patients, 49.5% had more than 20 teeth, while 13.4% had no remaining teeth. 116 patients wore some type of removable denture. Mandibular removable partial dentures were worn by 40 patients, and 31 patients had a maxillary removable partial dentures. The number of complete dentures was 121. The prosthetic index (the percentage of restored missing teeth) per person was 39.5%. The restorative index was 35.9%. Mucosal lesions were detected in 47 cases during the dental examination (11.5%). 103 patients (25.1%) had reported dental pain in the past 12 months. Necessity for any type of dental treatment was recognised in 271 patients (66.1%). 183 of patients (44.6%) had experiences self-reported xerostomia in the past 12 months.

### Dental status and socio-demographic factors

Table 2 shows the association between socio-demographic factors and dental status in patients after a stroke. The significance level was set at *p* < 0.05. However, the data obtained were even more significant and it was adopted (*p* < 0.0001).

**Table 2:** Socio-demographic factors associated with dental status

		D-T	M-T	F-T	DMFT
Factor	*n*	mean (SD)	mean (SD)	mean (SD)	mean (SD)
Gender					
Male	236	4.06 (4.38)^*a^	13.92 (10.17)^*a^	1.84 (2.97)	19.83 (7.99)
Female	174	2.22 (3.81)^*a^	16.50 (10.29)^*a^	1.82 (2.91)	20.53 (8.22)
Age groups (years)					
17–24	6	1.17 (1.33)^*b^	0.67 (1.63)^*b^	0.33 (0.82)^*b^	2.17 (1.33)^*b^
25–49	104	5.20 (4.72)^*b^	7.40 (6.50)^*b^	3.16 (3.58)^*b^	15.77 (6.57)^*b^
50–64	146	3.78 (4.23)^*b^	14.53 (9.46)^*b^	1.84 (2.92)^*b^	20.16 (7.64)^*b^
65–79	134	1.77 (3.38)^*b^	20.46 (9.03)^*b^	0.97 (1.95)^*b^	23.19 (7.20)^*b^
80–89	20	0.35 (0.93)^*b^	26.00 (8.68)^*b^	1.05 (2.98)^*b^	27.40 (5.92)^*b^
Location type					
Capital	154	2.67 (3.57)	15.12 (9.75)	2.07 (3.12)	19.86 (7.55)
Periurban area	39	3.13 (4.31)	15.23 (10.89)	1.79 (2.66)	20.15 (8.66)
Rural	217	3.74 (4.61)	14.91 (10.60)	1.67 (2.86)	20.31 (8.37)
Occupational status					
Unemployed	10	6.70 (4.06)^*b^	12.40 (6.52)^*b^	1.10 (1.85)^*b^	20.20 (6.44)^*c^
Employed	158	3.87 (4.40)^*b^	12.40 (9.51)^*b^	2.16 (3.10)^*b^	18.42 (7.68)^*c^
Self-employed	59	3.36 (3.42)^*b^	10.66 (9.01)^*b^	3.34 (3.72)^*b^	17.36 (6.94)^*c^
Housewife	4	7.75 (3.10)^*b^	9.00 (4.40)^*b^	0.50 (0.58)^*b^	17.25 (6.08)^*c^
Student	5	1.40 (1.34)^*b^	0.80 (1.79)^*b^	0.00 (0.00)^*b^	2.20 (1.48)^*c^
Stay at home parent	2	8.50 (12.02)^*b^	17.50 (3.54)^*b^	0.00 (0.00)^*b^	26.00 (8.49)^*c^
Pensioner	147	1.83 (3.36)^*b^	20.74 (9.70)^*b^	1.05 (2.14)^*b^	23.63 (7.40)^*c^
Altered work ability	25	5.76 (6.01)^*b^	12.84 (8.70)^*b^	1.80 (3.21)^*b^	20.40 (8.35)^*c^
Level of education					
No formal schooling	2	3.50 (4.95)^*b^	20.50 (16.26)^*b^	0.00 (0.00)^*b^	24.00 (11.31)^*c^
< Primary school	5	0.60 (0.89)^*b^	21.80 (10.47)^*b^	1.40 (2.07)^*b^	23.80 (7.95)^*c^
Primary school	54	4.04 (4.52)^*b^	18.98 (10.25)^*b^	0.83 (2.14)^*b^	23.85 (7.58)^*c^
Secondary school	92	3.98 (4.74)^*b^	16.37 (10.31)^*b^	1.32 (2.43)^*b^	21.66 (8.09)^*c^
High school	126	3.64 (4.42)^*b^	13.84 (9.81)^*b^	1.83 (2.83)^*b^	19.31 (7.83)^*c^
College/University	130	2.24 (3.39)^*b^	13.28 (10.17)^*b^	2.67 (3.49)^*b^	18.19 (7.81)^*c^
Postgraduate degree	1	0.00^*b^	5.00^*b^	1.00^*b^	6.00^*c^

*SD* Standard Deviation, *D-T* Number of decayed teeth, *M-T* Number of missing teeth; *F-T* Number of filled teeth, *DMFT* Sum of the number of decayed, missing and filled teeth

^*^p < 0.05 (^a^Mann-Whitney U test, ^b^Kruskal-Wallis test, ^c^ANOVA model)

Significant relationships were found between gender and D-T (*p* < 0.0001), M-T (*p* = 0.0085) values, while no connection was found between F-T (*p* = 0.6298) and DMFT (*p* = 0.3834) scores. Females had significantly fewer decayed teeth compared to males, while they had significantly more missing teeth. D-T, M-T, F-T and DMFT were related by age distribution. Significant differences were found between age groups and D-T, M-T, F-T and DMFT scores. The most decayed and filled teeth were in the groups of 25–49-year-olds, while the most missing teeth and the highest DMFT score were in 80–89-year-old patients. No significant correlation was found between D-T (*p* = 0.0829), M-T (*p* = 0.8643), F-T (*p* = 0.199) and DMFT (*p* = 0.8667) scores and location type (urban, periurban area, rural), but it was observed among the data that most people with carious teeth had a rural address. Significant differences were observed between occupational status and D-T, M-T, F-T, DMFT scores. Post-stroke patients, who were inactive, had the highest D-T (stay at home parent), M-T (pensioner), DMFT values (stay at home parent), while the highest F-T values were seen in the active group (self-employed). D-T, M-T, F-T and DMFT values showed significant relationships with level of education (*p* = 0.007, *p* = 0.0046, *p* = 0.0008, *p* < 0.0001, respectively). The highest D-T, M-T and DMFT scores were found among those with lower education (primary school completed, less than primary school) while those with the highest values for filled teeth had graduated from high school, college, or university. A detailed table of socio-demographic factors associated with dental status can be found in our Additional file 1.

### Dental status and stroke-related factors

The proportion of subarachnoid hemorrhage, intracerebral hemorrhage and cerebral infarction cases were 3.9%, 17.8% and 78.3%. The association of stroke types with dental status are summarized in Table 3.

**Table 3:** Association between stroke type and dental status

		D-T	M-T	F-T	DMFT
Stroke type	*N*	mean (SD)	mean (SD)	mean (SD)	mean (SD)
Subarachnoid hemorrhage	16	2.62 (3.48)	12.19 (8.63)^*^	2.19 (3.12)	17.00 (7.57)^*^
Intracerebral hemorrhage	73	3.48 (3.77)	12.32 (9.37)^*^	1.99 (2.84)	17.78 (7.41)^*^
Cerebral infarction	321	3.26 (4.38)	15.77 (10.46)^*^	1.78 (2.96)	20.82 (8.14)^*^

*SD* Standard Deviation, *D-T* Number of decayed teeth, *M-T* Number of missing teeth, *F-T* Number of filled teeth, *DMFT* Sum of the number of decayed, missing and filled teeth;

^*^p < 0.05 (Kruskal–Wallis test)

Significant relationships were found between DMFT value (*p* = 0.0025), M-T component (*p* = 0.0259) and stroke type, while no differences were observed D-T (*p* = 0.3148) and F-T (*p* = 0.5588) components. The highest DMFT and M-T score were recorded in cerebral infarction type. The lowest DMFT score were seen for the subarachnoid subtype. A detailed table of stroke type associated with dental status can be found in our Additional file 2. No significant differences were observed betweeen post-stroke symptoms (hemiplegia/hemiparesis, tetraplegia, dysphagia, dysarthria/anarthria, ataxia and depression) and DMFT scores. A detailed table of post-stroke symptoms associated with DMFT score can be found in our Additional file 3.

Among the risk factors for stroke (Table 4), significant differences were observed between hypertension and M-T score (*p* = 0.0106), alcohol consumption and M-T score (*p* = 0.02362), smoking and D-T score (*p* = 0.008). A detailed table of risk factors for stroke associated with dental status can be found in our Additional file 4.

**Table 4:** Risk factors for stroke associated with dental status in post-stroke patients

			D-T	M-T	F-T	DMFT
Risk factor	Yes/No	*n*	mean (SD)	mean (SD)	mean (SD)	mean (SD)
Diabetes mellitus	Yes	88	3.51 (4.20)	16.24 (10.13)	1.53 (2.48)	21.28 (7.84)
	No	322	3.21 (4.25)	14.68 (10.32)	1.91 (3.06)	19.81 (8.13)
Hypertension	Yes	310	3.25 (4.16)	15.61 (9.98)^*^	1.58 (2.71)	20.43 (7.72)
	No	100	3.37 (4.51)	13.19 (11.06)^*^	2.62 (3.47)	19.18 (9.10)
Hyperlipidemia	Yes	121	3.55 (4.43)	15.90 (10.34)	1.69 (2.97)	21.14 (7.72)
	No	289	3.16 (4.16)	14.65 (10.26)	1.89 (2.93)	19.70 (8.21)
Alcohol consumption^a^	Yes	138	3.31 (4.28)	16.38 (9.72)^*^	1.46 (2.51)	21.15 (7.77)
	No	272	3.26 (4.23)	14.33 (10.51)^*^	2.02 (3.13)	19.61 (8.21)
Smoking^b^	Yes	293	2.83 (3.83)^*^	15.03 (10.44)	1.96 (3.07)	19.82 (8.11)
	No	117	4.41 (4.96)^*^	14.98 (9.94)	1.50 (2.57)	20.90 (8.01)

*SD* Standard deviation, *D-T* Number of decayed teeth, *M-T* Number of missing teeth, *F-T* Number of filled teeth, *DMFT* Sum of the number of decayed, missing and filled teeth; ^*^p < 0.05 (Mann–Whitney *U* test)

^a^ Subcategories of alcohol consumption are created by aggregating the number of patients who reported to consume alcohol in the past 30 days

^b^ Subcategories of smoking are defined by aggregating the number of patients who reported either the permanent or occasional of any tobacco product (cigarettes, cigars, pipe, snuff)

**Fig. 2:**
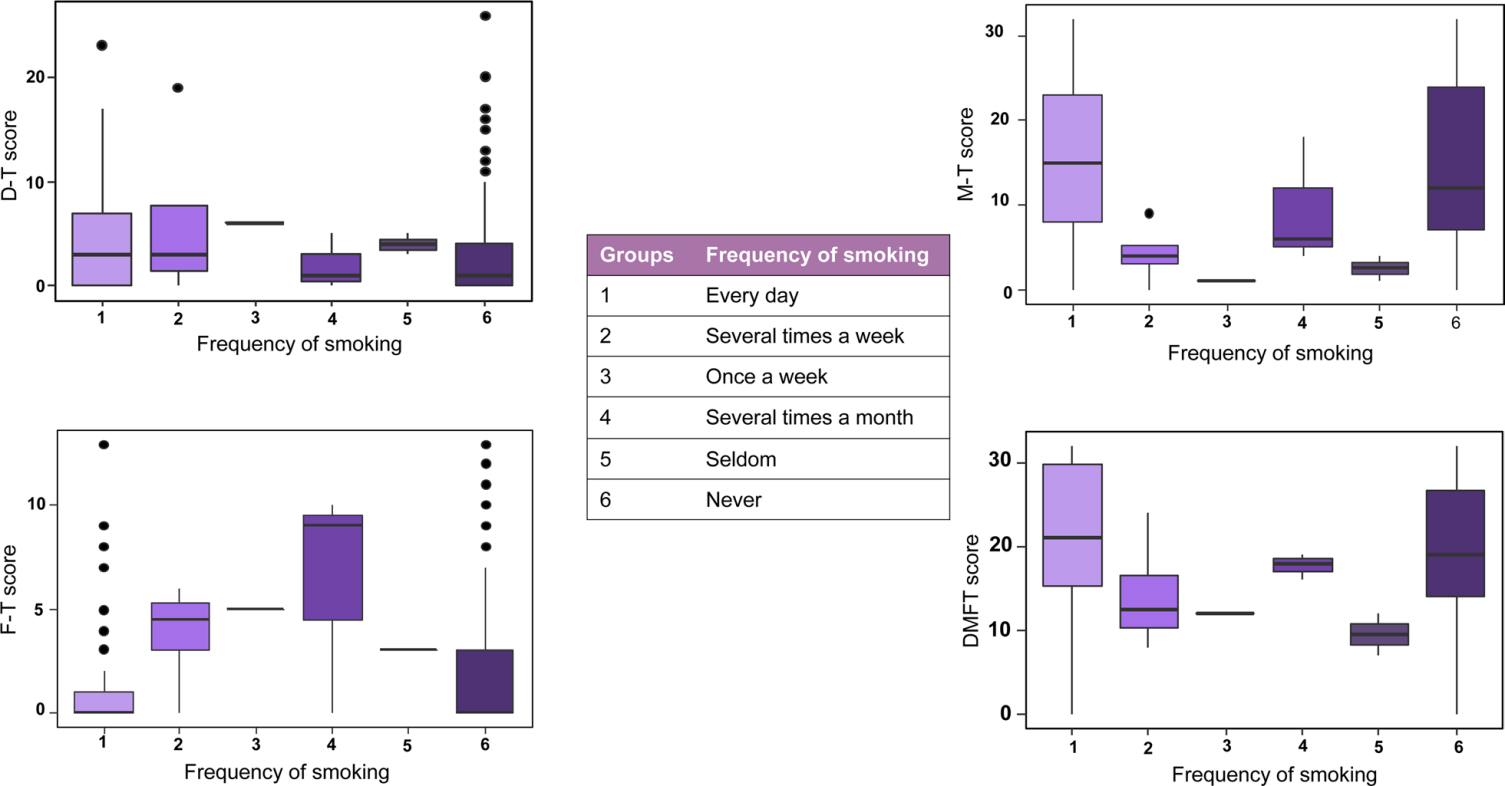
Frequency of smoking associated with dental status in post-stroke patients. D-T score: number of decayed teeth; M-T score: number of missing teeth; F-T score: number of filled teeth; DMFT score: sum of the number of decayed, missing and filled teeth

Figure 2 shows the association between frequency of smoking and dental status in patients after a stroke. The frequency of smoking was significantly associated with M-T (*p* = 0.0175) and F-T score (*p* = 0.0282). The highest mean M-T value was 15.89 (9.82) in the patient group where they smoked every day, while the highest mean F-T value was 6.33 (5.51) in the group where they smoked several times a month.

### Dental status and functional assessment

**Fig. 3:**
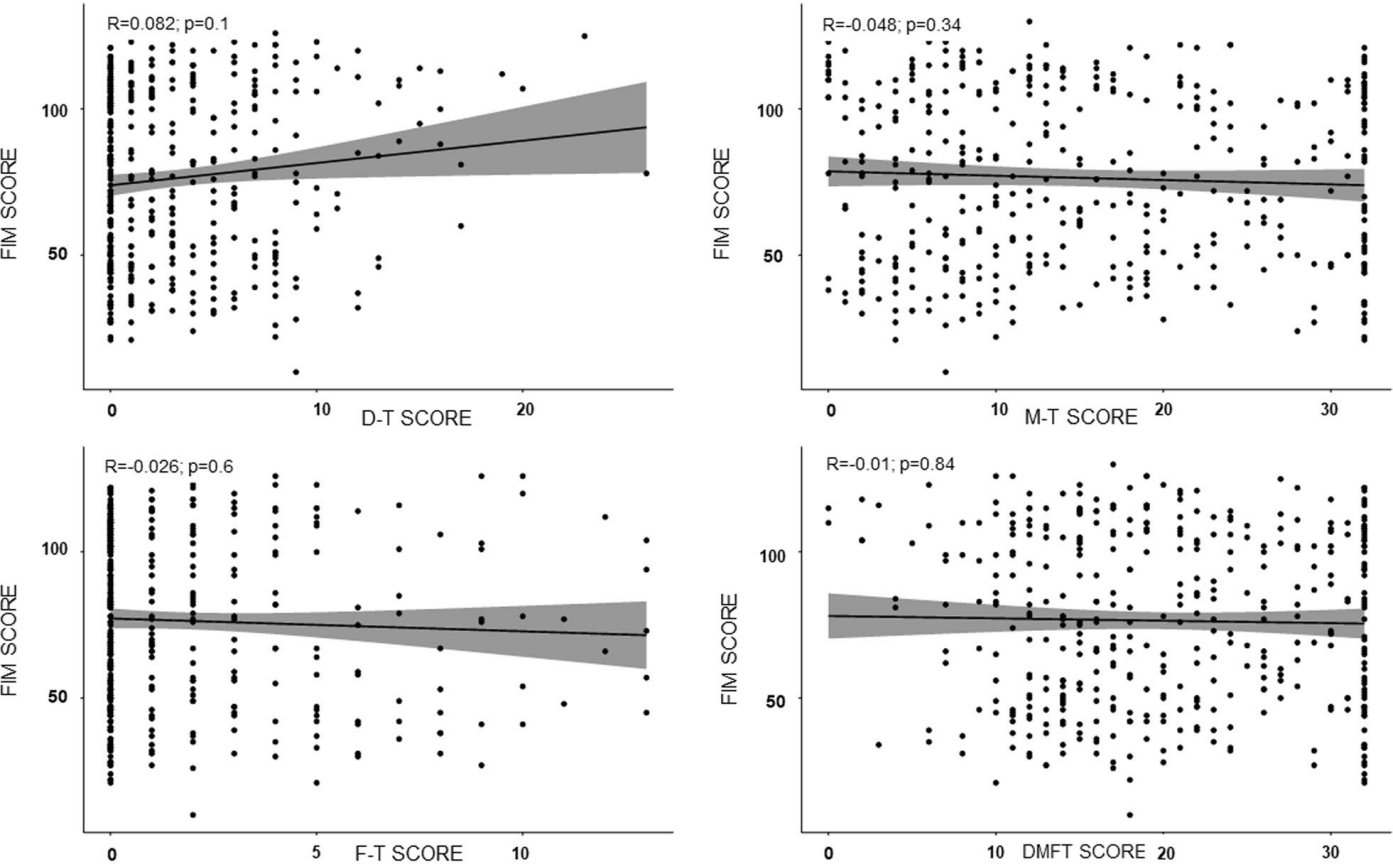
Association between dental status and Functional Independence Measure. *R*: correlation coefficient; *p*: *p*-value. FIM score: Functional Independence Measure score. D-T score: number of decayed teeth; M-T score: number of missing teeth; F-T score: number of filled teeth; DMFT score: sum of the number of decayed, missing and filled teeth.

**Fig. 4:**
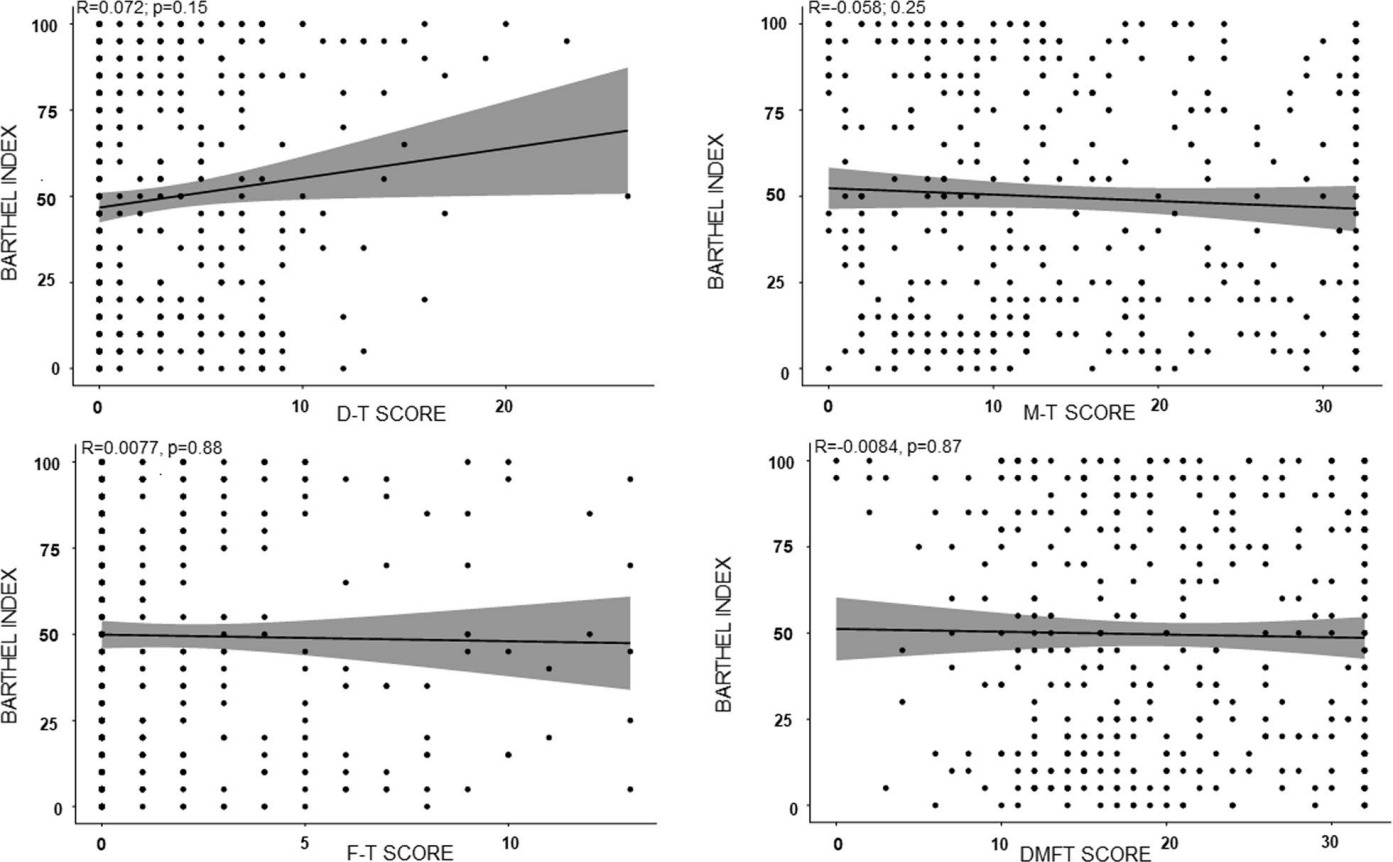
Association between dental status and Barthel index. *R*: correlation coefficient; *p*: *p*-value. D-T score: number of decayed teeth; M-T score: number of missing teeth; F-T score: number of filled teeth; DMFT score: sum of the number of decayed, missing and filled teeth

No significant correlation was found between FIM, Barthel index and D-T, M-T, F-T, DMFT scores. Figure 3 shows the association between dental status and FIM. A weak positive correlation was detected the FIM value and D-T scores (correlation coefficient [*R*] = 0.082), and a weak negative correlation for the M-T, F-T and DMFT values (*R* = − 0.048, *R* = − 0.026, *R* = − 0.01, respectively), both insignificant. Figure 4 shows the association between dental status and Barthel index. A weak positive correlation was detected for the D-T and F-T scores (*R* = 0.072, *R* = 0.0077, respectively) and a weak negative correlation for the M-T and DMFT values (*R* = − 0.058, R = − 0.0084, respectively), both insignificant.

### Oral health behavioural factors

**Fig. 5:**
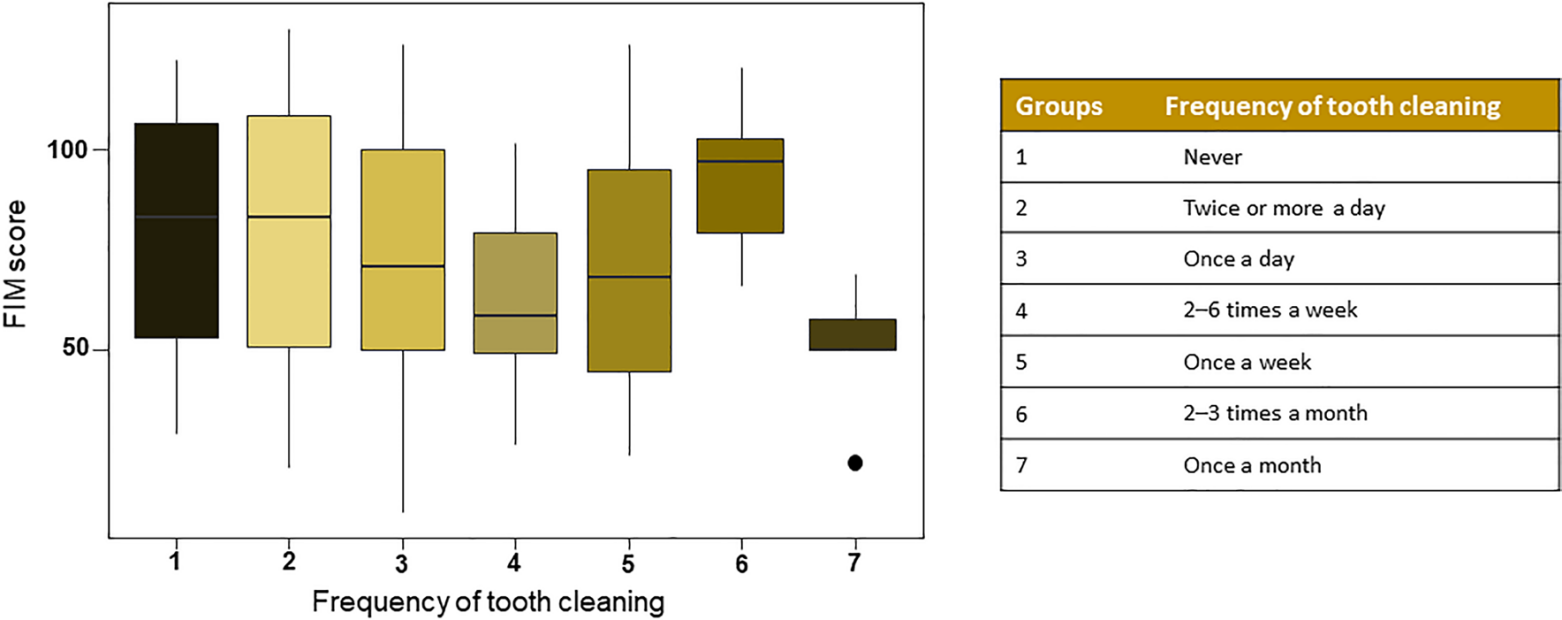
Frequency of tooth cleaning association with Functional Independence Measure. FIM score: Functional Independence Measure score

Table 5 shows the oral health behavioural characteristics of post-stroke patients. Only 69 patients (16.8%) reported attending to dental visit within six months. More than one fifth of patients (21.7%) had not undergone dental screening for more than 5 years. As for the frequency of tooth cleaning, 44.6% of the patients reported brushing their teeth twice or more a day. 172 (42%) post-stroke patients reported using a mouthwash and only 7 (1.7%) reported flossing. Out of 116 patients, who were wearing some form of removable denture, 70 of them (60.3%) responded that they use a denture cleaner. As for brushing frequency, Fig. 5 shows the association between tooth cleaning frequency and the FIM. Significant differences were found (*p* = 0.0402) in relation to the FIM value. Patients with the lowest mean FIM score (mean 49.80 (17.38)) were those who reported brushing their teeth once a month, while those who brushed their teeth two or more times a day had a mean FIM score of 80.39 (29.92).

**Table 5:** Oral health behavioural characteristics

Behavioural variables	Overall *n* = 410
Last dental visit, *n* (%)	
< 6 months	69 (16.8)
6–12 months	75 (18.3)
1–2 years	78 (19.0)
2–5 years	78 (19.0)
> 5 years	89 (21.7)
Never	3 (0.7)
Self-reported frequency of tooth cleaning, *n* (%)	
Twice or more a day	183 (44.6)
Once a day	164 (40.0)
2–6 times a week	10 (0.2)
Once a week	22 (5.4)
2–3 times a month	6 (1.5)
Once a month	6 (1.5)
Never	19 (4.6)
Device used for tooth cleaning, *n* (%)	
Manual toothbrush	303 (73.9)
Electric toothbrush	24 (5.9)
Electric and manual toothbrush	50 (12.2)
Does not use a toothbrush	33 (8.1)

## Discussion

Post-stroke patients undergoing rehabilitation are at risk of poor oral health for a number of reasons. The long-term effects of cerebral damage makes it difficult to maintain good oral health. This group of patients have worse oral health status across a range of parameters (tooth loss, dental caries, periodontal status, prosthetic index) compared to people who did not have a stroke [8–11].

In the present study, we assessed the oral health status of stroke patients undergoing rehabilitation treatment and explored the factors associated with dental status.

Dental health can be defined and measured by the score of dental caries intensity index (DMFT) and their components (D-T, M-T, F-T). The present research revealed high mean DMFT (20.13), D-T (3.28), M-T (15.02) scores, and low mean F-T (1.83) score. This result was worse compared with healthy Hungarian population [31]. Only one Hungarian study was examined the oral health status of post-stroke patients and was received lower mean D-T value (2.3 (3)) and higher F-T score (3.6 (4.7)) [8]. The prosthetic index (the percentage of restored missing teeth) was 39.5%, which is also very bad [8]. The restorative index was 35.9%, which means that many caries is unattended [8]. These results may be related to the fact that poor socio-demographic situation, physical disfunctions and difficulties in accessing dental facilities do not allow adequate restorative and prosthetic dental treatment. Unfortunately, in many cases, due to communication problems, mental health problems, lack of compliance dentist might choose treatment options that are simple and quick. Dental professionals are not trained to care for the special need population. In Hungary, there is no available guideline for the management of complex dental care for stroke patients. [21, 32]

The necessity of dental treatment was recognised in our study in 271 patients (66.1%), which could be due to the neglected oral health care of patients after stroke. Oral health care plays an important role in preventing oral disorders and serious complications such as pneumonia, plus increased length of hospital stay [14].

In the current study, the most important socio-demographic factors that influenced DMFT and their components were gender, age, occupational status and level of education. The majority of the participants in this study were male. It has been recognised long ago that stroke incidence is higher in males than in females globally [33]. Females after stroke had significantly fewer decayed teeth, compared to males, while they had significantly more missing teeth. Similar results in the Hungarian adult population were reported by Madléna et al. [31]. The most decayed and filled teeth were in the group 25–49-year-olds, while the most missing teeth and the highest DMFT score were in 80–89-year-olds patients. Low occupational status and low level of education was associated with poorer dental status (more decayed teeth, more missing teeth, highest DMFT score), while people with an active occupational status and with tertiary-level education reported the highest F-T (filled teeth) score. These results from a previous report confirmed that lower level occupational and educational status may have a negative effect on dental status [34, 35].

There are two main types of stroke ‒ ischemic, including transient ischemic, and hemorrhagic, including intracerebral and subarachnoid [36]. In the present study, subarachnoid, intracerebral and ischemic (cerebral infarction) stroke types were examined. Transient ischemic attack was not found in the study population under rehabilitation treatment, these patients do not require long-term rehabilitation treatment. More than three quarters of strokes were of the cerebral infarction type, which is even the most common cause of stroke [33]. Many researchers suggested an association between poor oral health and ischemic stroke [5, 37, 38]. And in fact, significant relationships were found between DMFT scores, M-T scores and this type of stroke. Patients with cerebral infarction had the highest number of missing teeth and DMFT score, while the lowest DMFT value was seen in the subarachnoid subtype. Similar results were found between the association of missing teeth and cerebral infarction by Alhadainy et al. [7] and Lee et al. [39]. The better DMFT value of the subarachnoid type can be explained by the fact that this type is mostly present in young adults [40]. The worst DMFT value for cerebral infarction may be due to the fact that oral health diseases and ischemic stroke have many factors in common, including old age, diabetes mellitus, tobacco smoking, alcohol consumption and low socio-economic status [41].

Post-stroke sequels constitute a substantial challenge in the daily oral health care routine and dental visits [11]. In the present study, the leading residual sequel was hemiplegia, that is a paralysis of half of the body, which can cause an inability to use the affected side. Mobility impairment makes it difficult to carry out optimal tooth brushing. Insufficient oral care leads to increased oral infections and dental caries [13]. Impairment in swallowing (dysphagia) is also a significant problem for post-stroke patients. Dysphagia and poor oral health status are risk factors for aspiration pneumonia [42]. However, the present study shows that no significant differences were between post-stroke symptoms and the DMFT score. This can be explained by the small number of patients diagnosed with dysphagia. Further examinations are necessary to investigate the combined effect of these symptoms on oral health.

The most important modifiable risk factors for stroke are well-documented and include hypertension, diabetes mellitus, hyperlipidemia, tobacco smoking and alcohol consumption are well-documented [4]. High blood pressure is the major risk factor for all stroke types [40]. And, in the current study, more than 75% of patients had hypertension. The results showed a significant association between hypertension and number of missing teeth. Patients with high blood pressure had higher M-T scores, compared to those who did not have. Periodontitis, which can lead to tooth loss and hypertension, share specific demographics and risk factors such as older age, male gender, smoking, obesity, diabetes mellitus, low socio-economic status, and poor education [43]. In light of this, management of traditional cardiovascular risk factors, including hypertension, is of great importance in the management of periodontitis. Besides, treating periodontitis is essential in order to achieve risk-free general health [44].

Although no significant difference was found between the dental status of diabetic and non-diabetic patients in the present study, the effect of diabetes on oral health is unquestionable. Diabetes has a great number of complications in the oral cavity, including dryness of the mouth, which may cause other problems, as increased caries activity, increased oral fungal infections, dysphagia, glossodynia [45].

There was a significant difference in D-T values between smoking (median D-T = 1) and non-smoking (median D-T = 3) patients, which means that smoking patients had fewer decayed teeth. The literature does not show a clear relationship between tobacco smoking and dental caries. The recent study demonstrated an decreased prevalence of caries in smokers. Similar results have been presented by Hugosan et al. [46]. Examining the frequency of smoking, it can be said that most of the missing teeth were among those who smoked every day. The destructive force of smoking against the periodontal tissues clinically manifest in several forms, as bone loss, attachment loss and finally tooth loss [47].

The role of alcohol consumption as a risk factor for stroke has been widely studied [48]. Alcoholics have higher risk of developing dental caries, gingival diseases and oropharyngeal cancers. Enberg et al. [49] found that the number of missing teeth were higher in alcoholics than non-alcoholics.

In the current study, the FIM was represented as a measure of disability and independence for self-care [23]. However no significant correlation was found between FIM and dental status, but frequency of teeth cleaning was related to FIM scores. Patients with the lowest FIM score (mean FIM = 49.80 (17.38)) were those who reported brushing their teeth once a month, while those who brushed twice or more a day had a mean FIM score of 80.39 (29.92). According to evidence based on oral health care, tooth brushing at least twice a day is one of the most essential practices for good oral hygiene [50]. In this research, only 44.6% of the patients reported the recommended brushing frequency. Oral hygiene routines may be disrupted by physical disabilities that may make oral self-care more difficult [51]. Patients with stroke are less frequent dental attenders [10]. In this study only 16.8% of post-stroke patients had received a full mouth examination in the last half year. 21.7% of patients have not undergone dental screening for more than 5 years. Patients with physical disabilities have limited access to dental care due to difficulty in finding barrier free dental offices [52].

## Strengths and limitations

The present study examined an important topic that is receiving increasing attention in the international literature. The location of the study was The National Institute for Medical Rehabilitation, which is a nation-wide rehabilitation centre. The sample size (*n* = 410), was enough to validate most of the conclusions. However, some limitations of this study should be mentioned. First is the lack of age- and sex-matched healthy control group. In the current research, the main aim was to assess the oral health status of stroke patients undergoing rehabilitation treatment and to explore diverse set of factors that may influence it. In a future study, we designed a case–control study to compare the oral health status between the healthy and post-stroke groups. In addition to the control group (people who did not have a stroke), there would also be a third group of patients who had a stroke and who are not undergoing rehabilitation. Second, the validity of the results for some subgroups can be limited due to low number of patients. Third, the oral hygiene status and the periodontal status were not assessed.

### Interpretation

Considering the limitations of the study, the proposed objectives were achieved. In summary, it can be stated that post-stroke patients had worse oral health status in a series of parameters (tooth loss, dental caries, restorative index and prosthetic index). Poor oral health was associated with several socio-demographic aspects (sex, age distribution, occupational status and education level), type of stroke, known risk factors (hypertension, smoking and alcohol consumption) and behavioural factors in oral health (frequency of dental cleaning and access to dental care). According to the data, a risk group, due to the high number of decayed teeth, is composed of young men, aged 25–49, unemployed, poor and smokers. Another risk group, due to the high number of missing teeth, is women over 80 years old, who have suffered ischemic stroke, pensioners, the poor, but who had access to education, and hypertensive and alcoholic individuals.

## Conclusion

Based on the results of this research, it can be said that post-stroke patients had a worse oral health status in a series of parameters. Poor oral health was correlated with several aspects: socio-demographic, type of stroke, risk and behavioural factors in oral health, combined with physical or cognitive difficulties, even though they are in the process of rehabilitation. According to the data, low socio-demographic and economic status, low level of education and FIM score, unemployment, the combination of risk factors for stroke and residual dysfunctions are associated with poor oral health status. Therefore, the findings point to structural changes and the need for special attention and oral health care for post-stroke individuals. A new model of prevention, care and promotion can be developed, at an individual and collective level, with an interdisciplinary partnership, in order to help adapt to new circumstances and restore, as far as possible, the quality of life of people after a stroke.

## Supplementary Information

**Additional file 1: **Socio-demographic factors associated with dental status.

**Additional file 2: **Association between stroke type and dental status.

**Additional file 3: **Post-stroke symptoms associated with DMFT in post-stroke patients.

**Additional file 4: **Risk factors for stroke associated with dental status in post-stroke patients.

**Additional file 5: **Raw data

## Data Availability

All data generated or analysed during this study are included in this published article and its supplementary information files.

## Abbreviations

ANOVA: Analysis of variance technique by blocks
DMFT: Decayed (D-T), Missing (M-T) and Filled (F-T) Teeth index
FIM: Functional independence measure
ICD-10: International classification of diseases, 10th revision
NIMR: National Institute for Medical Rehabilitation
SD: Standard deviation
STROBE: Strengthening the reporting of observational studies in epidemiology
R: Correlation coefficient
WHO: World Health Organization

## References

[1] Mackay J, Mensah GA (2004). The atlas of heart disease and stroke.

[2] Hungarian Central Statistical Office. https://www.ksh.hu/stadat_files/ege/hu/ege0024.html Accessed 5 Aug 2021

[3] Hankey GJ (2017). Stroke Lancet.

[4] Johansson A, Drake I, Engström G, Acosta S (2021) Modifiable and Non-Modifiable Risk Factors for Atherothrombotic Ischemic Stroke among Subjects in the Malmö Diet and Cancer Study. Nutrients 13(6): 1952. 10.3390/nu13061952 PMC8229981 34204127

[5] Pillai RS, Iyer K, Spin-Neto R, Kothari SF, Nielsen JF, Kothari M (2018). Oral health and brain injury: causal or casual relation?. Cerebrovasc Dis Extra.

[6] Chang Y, Woo HG, Lee JS, Song TJ (2021). Better oral hygiene is associated with lower risk of stroke. J Periodontol.

[7] Alhadainy HA, Keefe T, Abdel-Karim AH, Abdulrab S, Halboub E (2021). Association between dental diseases and history of stroke in the United States. Clin Exp Dent Res.

[8] Karolyhazy K, Aranyi Z, Hermann P, Vastagh I, Marton K (2018). Oral health status of stroke patients related to residual symptoms: a case-control epidemiological study in Hungary. Oral Health Prev Dent.

[9] Zeng LN, Rao WW, Luo SH, Zhang QE, Hall BJ, Ungvari GS (2020). Oral health in patients with stroke: a meta-analysis of comparative studies. Top Stroke Rehabil.

[10] Dai R, Lam OL, Lo EC, Li LS, Wen Y, McGrath C (2015). A systematic review and meta-analysis of clinical, microbiological, and behavioural aspects of oral health among patients with stroke. J Dent.

[11] Kothari M, Pillai RS, Kothari SF, Spin-Neto R, Kumar A, Nielsen JF (2017). Oral health status in patients with acquired b. Oral Surg Oral Med Oral Pathol Oral Radiol.

[12] Kim HT, Park JB, Lee WC, Kim YJ, Lee Y (2018). Differences in the oral health status and oral hygiene practices according to the extent of post-stroke sequelae. J Oral Rehabil.

[13] Kwok C, McIntyre A, Janzen S, Mays R, Teasell R (2015). Oral care post stroke: a scoping review. J Oral Rehabil.

[14] Shiraishi A, Yoshimura Y, Wakabayashi H, Tsuji Y (2017). Poor oral status is associated with rehabilitation outcome in older people. Geriatr Gerontol Int.

[15] Gurgel-Juarez N, Perrier MF, Hoffmann T, Lannin N, Jolliffe L, Lee R (2020). Guideline recommendations for oral care after acquired brain injury: protocol for a systematic review. JMIR Res Protoc.

[16] Bangee M, Martinez-Garduno CM, Brady MC, Cadilhac DA, Dale S, Hurley MA (2021). Oral care practices in stroke: findings from the UK and Australia. BMC Nurs.

[17] British Society of Gerodontology. Guidelines for the oral healthcare of stroke survivors. 2010. https://www.gerodontology.com/content/uploads/2014/10/stroke_guidelines.pdf Accessed 25 Mar 2022.

[18] Orsos M, Moldvai J, Kivovics P, Nemeth O (2018). Oral health related quality of life of patients undergoing physical medicine and rehabilitation. Orv Hetil.

[19] Moldvai J, Orsós M, Simon F (2019). Descriptive study of oral health, dental care and health behavior of inpatients undergoing physical medicine and rehabilitation. Oral Health Care.

[20] World Medical Association. WMA Declaration of Helsinki – ethical principles for medical research involving human subjects. https://www.wma.net/policies-post/wma-declaration-of-helsinki-ethical-principles-for-medical-research-involving-human-subjects/ Accessed 26 Mar 2022

[21] Orsós M, Moldvai J, Simon F, Putz M, Merész G, Németh O (2021). Oral health status of physically disabled inpatients - results from a hungarian single-centre cross-sectional study. Oral Health Prev Dent.

[22] International statistical classification of diseases and related health problems. 10th Revision. 2019. https://icd.who.int/browse10/2019/en#/ Accessed 6 Sep 2021

[23] Linacre JM, Heinemann AW, Wright BD, Granger CV, Hamilton BB (1994). The structure and stability of the functional independence measure. Arch Phys Med Rehabil.

[24] Mahoney FI, Barthel DW (1965). Functional evaluation: The barthel index. Md State Med J.

[25] Prodinger B, O'Connor RJ, Stucki G, Tennant A (2017). Establishing score equivalence of the functional independence measure motor scale and the barthel index, utilising the international classification of functioning, disability and health and rasch measurement theory. J Rehabil Med.

[26] Brown AW, Therneau TM, Schultz BA, Niewczyk PM, Granger CV (2015). Measure of functional independence dominates discharge outcome prediction after inpatient rehabilitation for stroke. Stroke.

[27] Physiopedia: Barthel Index. https://www.physio-pedia.com/Barthel_Index Accessed 3 Jan 2022

[28] Hungarian Central Statistical Office. https://www.ksh.hu/docs/hun/xstadat/xstadat_evkozi/15_64_abra_2012r.pdf Accessed 21 Sep 2021

[29] World Health Organization. Oral health surveys-basic methods. 5th ed. Geneva: World Health Organization; 2013. https://apps.who.int/iris/bitstream/handle/10665/97035/9789241548649_eng.pdf?sequence=1&isAllowed=y Accessed 28 Sep 2021

[30] Karolyhazy K, Kovacs E, Kivovics P, Fejerdy P, Aranyi Z (2003). Dental status and oral health of patients with epilepsy: an epidemiologic study. Epilepsia.

[31] Madlena M, Hermann P, Jahn M, Fejerdy P (2008). Caries prevalence and tooth loss in Hungarian adult population: results of a national survey. BMC Public Health.

[32] Sen S, Giamberardino LD, Moss K, Morelli T, Rosamond WD, Gottesman RF (2018). Periodontal disease, regular dental care use, and incident ischemic stroke. Stroke.

[33] Bushnell CD, Chaturvedi S, Gage KR, Herson PS, Hurn PD, Jimenez MC (2018). Sex differences in stroke: Challenges and opportunities. J Cereb Blood Flow Metab.

[34] Paulander J, Axelsson P, Lindhe J (2003). Association between level of education and oral health status in 35-, 50-, 65- and 75-year-olds. J Clin Periodontol.

[35] Al-Sudani FY, Vehkalahti MM, Suominen AL (2016). Association of current employment status with oral health-related behaviors: findings from the Finnish Health 2000 Survey. Eur J Oral Sci.

[36] Yan LL, Li C, Chen J, et al. In: Prabhakaran D, Anand S, Gaziano TA, Mbanya JC, Wu Y, Nugent R (eds) Cardiovascular, Respiratory, and Related Disorders. 3rd ed. Washington (DC): The international bank for reconstruction and development / The World Bank; 2017, Chapter 9. 30212054

[37] Leira Y, Seoane J, Blanco M, Rodriguez-Yanez M, Takkouche B, Blanco J (2017). Association between periodontitis and ischemic stroke: a systematic review and meta-analysis. Eur J Epidemiol.

[38] Sinha RK, Singh A, Kishor A, Richa S, Kumar R, Kumar A (2021). Evaluation of oral hygiene status in patients with hemorrhagic and ischemic stroke. J Pharm Bioallied Sci.

[39] Lee HJ, Choi EK, Park JB, Han KD, Oh S (2019). Tooth loss predicts myocardial infarction, heart failure, stroke, and death. J Dent Res.

[40] Smajlovic D (2015). Strokes in young adults: epidemiology and prevention. Vasc Health Risk Manag.

[41] Joshipura K (2002). The relationship between oral conditions and ischemic stroke and peripheral vascular disease. J Am Dent Assoc.

[42] Ortega O, Parra C, Zarcero S, Nart J, Sakwinska O, Clave P (2014). Oral health in older patients with oropharyngeal dysphagia. Age Ageing.

[43] Del Pinto R, Pietropaoli D, Munoz-Aguilera E, D'Aiuto F, Czesnikiewicz-Guzik M, Monaco A (2020). Periodontitis and hypertension: is the association causal?. High Blood Press Cardiovasc Prev.

[44] Sanz M, Marco Del Castillo A, Jepsen S, Gonzalez-Juanatey JR, D'Aiuto F, Bouchard P (2020). Periodontitis and cardiovascular diseases: Consensus report. J Clin Periodontol.

[45] Tavares M, Lindefjeld Calabi KA, San ML (2014). Systemic diseases and oral health. Dent Clin North Am.

[46] Hugoson A, Hellqvist L, Rolandsson M, Birkhed D (2012). Dental caries in relation to smoking and the use of Swedish snus: epidemiological studies covering 20 years (1983–2003). Acta Odontol Scand.

[47] Bergstrom J (2004). Tobacco smoking and chronic destructive periodontal disease. Odontology.

[48] Kuklina EV, Tong X, George MG, Bansil P (2012). Epidemiology and prevention of stroke: a worldwide perspective. Expert Rev Neurother.

[49] Enberg N, Wolf J, Ainamo A, Alho H, Heinala P, Lenander-Lumikari M (2001). Dental diseases and loss of teeth in a group of Finnish alcoholics: a radiological study. Acta Odontol Scand.

[50] Hitz Lindenmüller I, Lambrecht JT (2011). Oral care. Curr Probl Dermatol.

[51] O'Malley L, Powell R, Hulme S, Lievesley M, Westoby W, Zadik J (2020). A qualitative exploration of oral health care among stroke survivors living in the community. Health Expect.

[52] D'Addazio G, Santilli M, Sinjari B, Xhajanka E, Rexhepi I, Mangifesta R (2021). Access to dental care-a survey from dentists people with disabilities and caregivers. Int J Environ Res Public Health.

